# Factors Affecting Length of Stay for Observation Patients

**DOI:** 10.7759/cureus.4547

**Published:** 2019-04-26

**Authors:** Rachel S Dada, Anupam A Sule

**Affiliations:** 1 Internal Medicine, St. Joseph Mercy Oakland Hospital, Pontiac, USA

**Keywords:** length of stay (los), outcomes research, observation unit, case management, physician advisor, clinical decision unit

## Abstract

Objectives

The objective of this study was to determine whether the addition of a case manager and a physician advisor to the observation unit would decrease the length of stay (LOS) of observation patients.

Study design

This retrospective, observational study for observation patients was conducted in 2017.

Methods

At a tertiary-care, medium-sized, urban, community hospital, the LOS for all observation patients in 2017 (2, 981 clinical decision unit [CDU] patients and 1,248 non-cohort patients) was studied. Interventions studied were the addition of unit-based case manager and physician advisor to observation patient treatment teams.

Results

Patients assigned to the CDU had a shorter LOS than scattered patients, *p *< 0.0005. After the data was controlled for changes in LOS on inpatients using analysis of covariance (ANCOVA), none of the interventions resulted in statistically significant effects on LOS for CDU or scattered patients. Season, day of the week, the month of the year, and the presence of residents/medical students did not have any effect on LOS. Patients arriving at night had significantly shorter LOS than those arriving during the day or evening, *p *= 0.035 and *p *= 0.029, respectively.

Conclusions

Placing observation patients in a single unit is effective for decreasing LOS. The addition of case managers or physician advisors may not be an effective strategy to address the LOS. The presence of trainees does not hinder patient flow.

## Introduction

Observation care has been defined by the Centers for Medicare & Medicaid Services (CMS) as a specific, defined set of clinically appropriate services, which include ongoing assessment and reassessment and short-term treatment [1]. Patients under observation may either be sequestered in a designated location (referred to as clinical decision unit or “CDU” henceforth) or be assigned a bed anywhere in the hospital (referred to as “scattered” henceforth). The prevalence of CDUs has been progressively increasing in recent years. Despite the growing number of hospitals with CDUs, nearly two-thirds of the US hospitals lack CDUs [2-3]. Studies have demonstrated that patients treated in CDUs have shorter lengths of stay (LOS) when compared with scattered patients [2-4].

Assignment of unit-specific case managers for the inpatient population has been increasing in recent decades. The inclusion of a case manager in interdisciplinary team rounds has been shown to reduce LOS and facilitate cost-effective, coordinated patient care [5-7]. Physician advisors provide education to hospital staff regarding patient status, ensure completion of proper documentation, and expedite patient transitions of care [8]. Physician advisors work closely with case managers to facilitate appropriate utilization of resources for a hospitalized patient and to expedite safe discharge when medically ready. Data regarding the effect of case managers and physician advisors on observation patients LOS has not been published.

This study looked at various factors affecting the LOS of observation patients. Our null hypotheses were that the addition of a case manager to the CDU team, the addition of a physician advisor who took part in daily, in person rounds, and the addition of physician advisor electronic chart review will not affect LOS of observation patients.

## Materials and methods

Study design

A retrospective electronic medical record-based data analysis of all patients admitted under observation status at a tertiary care, medium-sized, urban community hospital between 01/01/2017 and 12/31/2017 (study group) was carried out. Data from all observation patients in 2016 and all inpatients in 2017 served as control data for covariate analysis.

The data elements collected included LOS from presentation to ER to time of discharge order, date of admission, date of discharge, location of the patient, disposition at discharge (skilled nursing facility, home, home with home care), status at discharge (changed to inpatient or discharged as observation), name of the attending, and number of consultations. The names of patients and electronic medical identifiers were not made available to the research team and patient charts were not accessed by the research team. IRB approval number: 2018039.

Interventions

Quarter 1 (January-March) served as base data with no changes where the CDU patients were managed by the emergency department case manager on an as-needed basis and the scattered patients by the unit case managers. Quarter 2 (April-June) involved the assignment of a case manager to the CDU. In quarter 3 (July-September), a physician advisor reviewed the EMR of all observation patients every day and met the case managers for all the observation patients (CDU as well as scattered) to provide strategies to expedite the pace of care. Finally, in quarter 4 (October-December), there was a substitution of in-person rounds for an electronic chart review by a physician advisor with communication with observation patient’s case manager via telephone.

Statistical analysis

Descriptive statistics were calculated. For univariate analyses, associations between categorical variables were examined using chi-squared tests. Correlations between continuous variables were assessed using Pearson’s correlation. Differences between groups on continuous variables were measured using *t*-tests and/or analysis of variance (ANOVA), followed by Scheffe's post hoc *t*-tests. For multivariate analysis, ANCOVA was used to see if the factors identified in the univariate analyses remained significant when controlling for inpatient length of stay. *P-* values <0.05 were considered significant. All analyses were performed using SPSS version 22 software. Graphs were plotted using Excel and means were plotted with error bars signifying the standard error.

To detect a change in LOS from 40 +/- 20 hours to 35 +/- 20 hours (mean +/- SD), at least 253 patients would be needed in each group, for 80% power and alpha = 0.05.

## Results

Effect of patient volume, season, inpatient changes, and cohorting

There was no correlation between the observation patients' daily LOS and the number of in-patients being discharged on that same day (Pearson correlation *r *= 0.02, *p* = 0.31). Similarly, there was no correlation between daily LOS and the number of discharges on that day in observation patients (2016); *r* = 0.02, *p *= 0.30.

**Figure 1:**
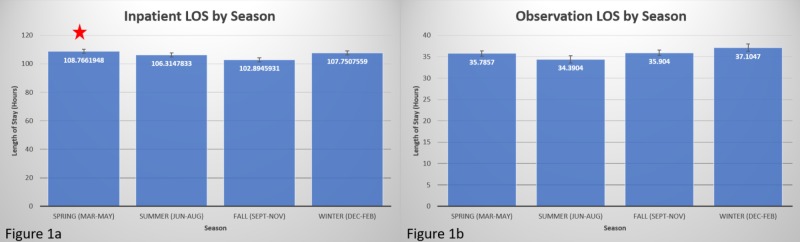
Variation in length of stay by season 1a. Inpatient LOS was significantly longer in spring (red star) compared to fall. 1b. Observation LOS was not significantly different in any season.

Patients were divided by season (spring: March-May; summer: June-August; fall: September-November; winter: December-February), and the mean LOS was compared for all seasons. In the inpatient population (Figure 1a), spring had a significantly longer LOS (mean: 108.76 hours, SD: 98.18 hours) than fall (mean: 102.89 hours, SD: 99.85 hours); *p *= 0.047. Summer (mean: 106.31 hours, SD: 100.76 hours) and winter (mean: 107.75 hours, SD: 100.1 hours) were not significantly different from the other seasons. For the observation population (Figure 1b), there was no significant seasonal variation in LOS. Spring (mean 35.78 hours, SD: 20.38 hours), summer (mean: 34.39 hours, SD: 27.67 hours), fall (mean: 35.90 hours, SD: 20.11 hours), and winter (mean: 37.10 hours, SD: 28.49 hours) had similar LOS; *p* = 0.091.

**Figure 2:**
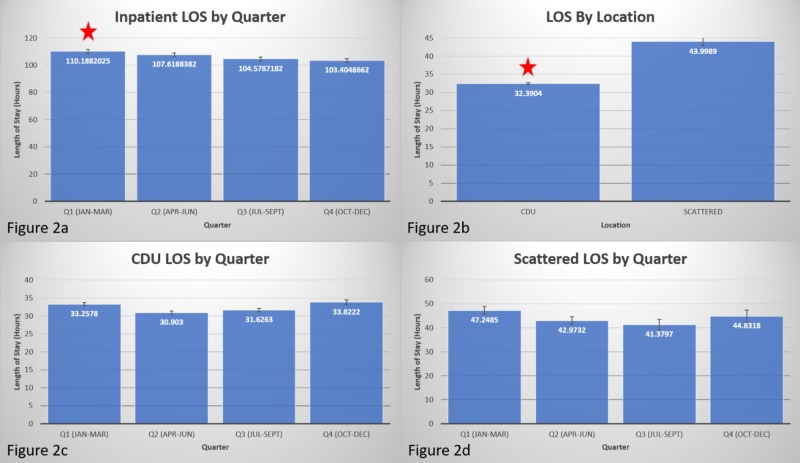
LOS by quarter Inpatient LOS was significantly longer in Q1 (red star) than in Q4. 2b. LOS for patients in CDU was significantly shorter than those scattered to other locations. 2c. There was no significant difference in LOS in CDU in any quarter. 2d. There was no significant difference in LOS in scattered locations in any quarter. LOS, length of stay; CDU, clinical decision unit

Inpatients in Q1 had a significantly longer LOS (mean: 110.19 hours, SD: 100.68 hours) compared with inpatients in Q4 (mean: 103.40 hours, SD: 96.90 hours); *p *= 0.014 (Figure 2a). Inpatient LOS was used as a covariate for analysis of observation patient LOS data. Patients assigned to the CDU had a shorter LOS (mean: 32.39 hours, SD: 14.56 hours) than scattered patients (mean: 44.00 hours, SD: 37.80 hours); *p *< 0.0005 (Figure 2b).

Effect of case manager and physician advisor

For CDU (Figure 2c), the mean LOS was 33.26 hours with an SD of 14.60 hours at baseline in Q1 of 2017. Initially, there was a decrease in patient LOS in Q2 of 2017 in CDU (mean: 30.90 hours, SD: 13.25 hours). This increased slightly in Q3 and the mean was 31.62 hours and SD was 14.13 hours. A further increase was noted in Q4 with a mean of 33.82 hours and an SD of 16.07 hours.

For scattered (Figure 2d), the mean LOS was 47.25 hours with an SD of 28.19 hours. A decrease was seen in Q2 (mean: 42.97 hours, SD: 24.57 hours). Q3 mean was further decreased to 41.38 hours with an SD of 41.73 hours. Q4 mean increased to 44.83 hours with an SD of 47.28 hours.

When the data was controlled for changes in LOS on inpatients using ANCOVA, none of the changes in CDU or scattered were statistically significant.

Effect of discharge location and the need of subsequent inpatient care

**Figure 3:**
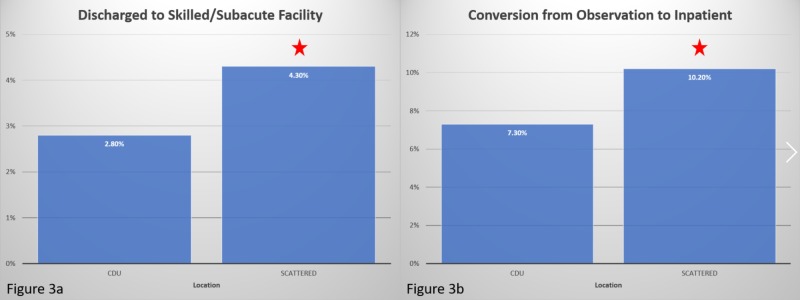
Outcomes versus location 3a. A significantly greater number of patients were discharged to skilled nursing facilities from scattered locations than CDU. 3b. A significantly greater number of patients were converted to inpatient from scattered locations compared to CDU. CDU, clinical decision unit

Patients discharged home with or without home health care had a significantly shorter LOS (mean: 35.21 hours, SD: 23.84 hours) than those discharged to a skilled nursing facility (mean: 51.26 hours, SD: 28.61 hours); *p *< 0.0005. Significantly fewer patients were discharged to a skilled nursing facility from the CDU (2.80%) compared to from the scattered (4.30%); *p *= 0.018. (Figure 3a) A significantly greater number of patients needed inpatient admissions from scattered locations (10.20%) compared to those from the CDU (7.30%); *p *< 0.0005 (Figure 3b).

Effect of temporal variation, consultation, and learners

**Figure 4:**
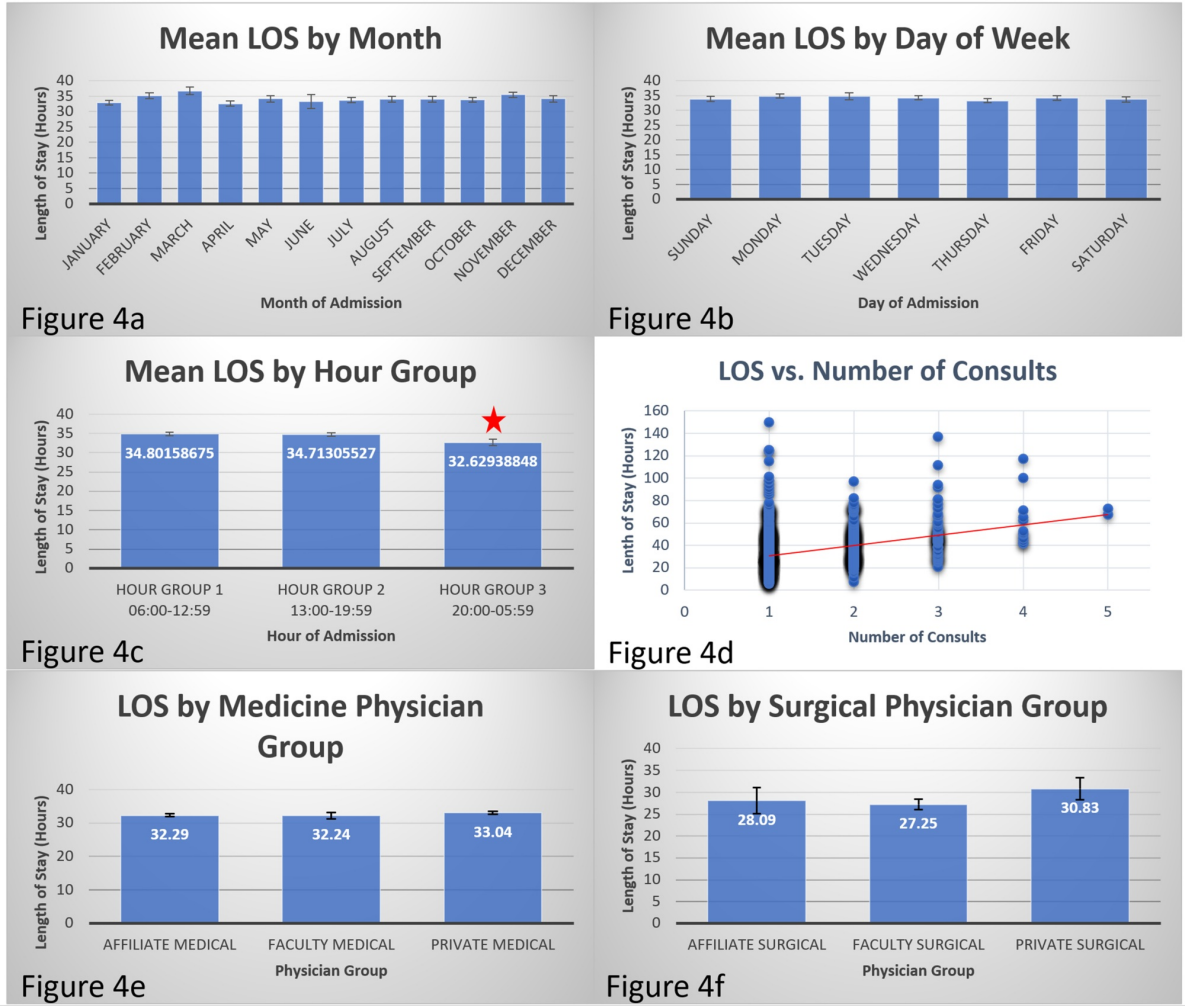
Various factors affecting LOS 4a. There was no significant difference in LOS by month. 4b. There was no significant difference in LOS by day of the week. 4c. Patients presenting at night had significantly shorter LOS (red star) compared to those arriving in the day. 4d. More number of consultations correlated to longer LOS. Figure 4e. The presence of learners did not impact LOS in medical patients. 4f. The presence of learners did not impact the LOS in surgical patients. LOS, length of stay

There was no difference in the LOS by day of the week (Figure 4a). On similar lines, there was no difference in the LOS by the month of the year (Figure 4b). Patients arriving during the day shift from 6:00 to 12:59 (mean: 34.80 hours, SD: 17.26 hours) and the afternoon shift from 13:00 to 19:59 (mean: 34.71 hours, SD: 19.35 hours) had significantly longer LOS compared to those arriving after hours from 20:00 to 05:59 (mean: 32.63 hours, SD: 28.31 hours); *p* = 0.035 and *p* = 0.029, respectively (Figure 4c). There was a weak but significant correlation between the number of consultations and LOS in the CDU (Pearson's correlation coefficient = 0.323, *p *< 0.0005; Figure 4d). There was no difference in the LOS whether residents and students on the internal medicine service were involved in the patient’s care throughout their stay (faculty medical, mean: 32.24 hours, SD: 15.00 hours), just for the initial placement in observation (affiliate medical, mean: 32.29 hours, SD: 15.27 hours), or not involved at all (private medical, mean: 33.04 hours, SD: 13.92 hours; Figure 4e). The same held true on the surgical service as well: faculty surgical (mean: 27.25 hours, SD: 12.88 hours), affiliate surgical (mean: 28.09 hours, SD: 13.25 hours), and private surgical (mean: 30.83 hours, SD: 12.88 hours) were not significantly different (Figure 4f).

## Discussion

Patients are placed under observation status when they do not meet the admission criteria at the time of presentation but are not appropriate for discharge either. The observation is expected to be completed within 24 hours [1-2]. In our study, 2,981 patients (70.49%) were placed in the CDU for observation, and 1,248 patients (29.51%) scattered to open hospital beds.

Forster et al. demonstrated that ED patient LOS is significantly increased when hospital occupancy exceeds 90% [9]. The observation LOS was neither affected by the number of inpatients nor observation patients, being discharged on the same day. This implied that the pace of care of observation patients was not slowed by a larger volume of discharges in the hospital or observation unit. Occupancy data was not available for correlation with LOS. Hultman et al. demonstrated longer hospital LOS for patients during the summer and winter months compared to fall and spring [10]. In our study, the LOS for patients admitted to the hospital in spring was significantly longer than fall, possibly due to the increase in respiratory allergens observed in spring in Michigan. There was no significant seasonal variation in the LOS for observation patients.

In an effort to decrease LOS, multiple changes were implemented in the hospital. This led to a steady improvement in the LOS of admitted inpatients with Q4 LOS being significantly shorter than Q1. To account for this confounding factor, inpatient LOS was used as a covariate during statistical analysis of observation patients LOS.

In 1996, 99% of patients in the CDU were discharged by the second day, whereas only 77% of the scattered patients were discharged by the second day [11]. A systematic review discovered that there was a significant reduction in LOS in short-stay units [12]. A retrospective analysis of LOS for observation patients revealed a decreased LOS following CDU implementation (2.4 days to 2.2 days, *p* = 0.05) [13]. In a national survey of United States observation units in 2003, Mace et al. estimated the mean LOS for CDU was 15.3 hours [14]. In an analysis of observation units in 2010, the average LOS was 17.2 hours [15]. In our study, patients assigned to the CDU (mean: 32.39 hours) had significantly shorter LOS than those scattered to an in-patient hospital bed (mean: 44.00 hours). An important distinction is that LOS in prior studies was from the time of admission to the observation unit while we considered the time from presentation to the ER.

A potential confounding factor in this comparison was the possibility that sicker patients were being sent to scattered location. A significantly greater number of patients needed inpatient admissions from scattered locations (10.20%) compared to those from the CDU (7.30%). Since the patients were in observation status, a case mix index was not generated and could not be compared. Patients whose status changed to inpatient were not included in the study. Another potential confounding factor was that significantly more patients from scattered location (4.30%) needed placement in a skilled nursing facility compared to CDU (2.80%) and those needing placement in the skilled nursing facility had a longer LOS (mean 51.26 hours) than those discharged home (mean 35.21 hours). If patients discharged to skilled nursing facilities were removed from the study group, the mean LOS for CDU decreased to 32.06 hours (SD = 13.74 hours), while that for scattered decreased to 43.04 hours (SD = 37.77 hours) and the difference was still statistically significant (*p* < 0.001). This confirms the effectiveness of CDU in decreasing the LOS.

In a review of 15 studies of inpatients, nine reported impact of case managers on LOS, and seven reported a reduced LOS [7]. Thomas et al. reported a decrease of one day in the median LOS with interdisciplinary rounding [8]. Hickey et al. reported that patients had 2.9 days shorter LOS when case managers were involved [5]. The effect of case managers in CDU has not yet been reported. Although a shorter LOS was observed in the CDU with a unit-based case manager compared to Q1, this was not statistically significant when controlled for changes in LOS for in-patients. The scattered observation LOS reflected a similar trend suggesting that the CDU case manager was not responsible for the decrease in LOS observed from Q1 to Q2. Also, this decrease in LOS could not be sustained and the LOS gradually lengthened as the year progressed. This may be due to the fact that most observation patients have fewer needs with respect to the transition of care. Thus, it might be more prudent to use case managers on a case by case basis in the CDU for those patients who have higher needs.

There is no data about the effect of physician advisors on observation LOS. There was no significant difference in the LOS for the patients in both CDU as well as scattered with either in person or electronic physician advisor participation. This reinforces the critical role of the attending physician in expediting LOS. Another physician providing suggestions to expedite the pace of care is not effective.

Hospitals are typically short staffed on the weekends and patient flow tends to slow down [16-17]. There was no difference in the LOS by day of the week. On similar lines, there was no difference in the LOS by the month of the year. The time of the day at which the patient was admitted to the CDU was a significant factor with those patients arriving after midnight having a shorter LOS compared to those arriving in the morning or afternoon. This was possibly due to the fact that the attending physicians rounded only once a day at our hospital. Patients arriving at night were seen in the morning and a plan of care was formulated early. Patients arriving after the attending physicians had finished rounding and left for the day experienced a delay in the formulation of their plan. If an attending physician sought more consultations, the patient had a longer LOS.

Historically it has been assumed that the presence of residents slows things down. Myers et al. observed that the median LOS for non-teaching service managing chest pain was 23 hours compared to 32 hours for teaching service [18]. The presence of residents did not hinder the pace of care in our study. Patients on both the medical and surgical services that were cared for by residents and medical students throughout their stay were compared to those seen just for initial admit orders, and those patients where residents were not involved in the patients’ care had similar LOS in the CDU.

The main challenge with the study was to isolate the effect of these specific interventions since multiple interventions were implemented to decrease LOS throughout the year by the hospital. Using the inpatient LOS as a covariate in ANCOVA may account for most of these changes but may not have eliminated all factors.

In light of the information gleaned from the above study, potential strategies that might reduce the LOS include multidisciplinary rounds with a single attending physician responsible for the care of all the patients in the unit as an effective and efficient way to address patient status, progress, and future goals [6,19]. Although the success of unit rounds has been demonstrated for the inpatient setting, in order for unit rounds to succeed for observation patients, the frequency of unit rounds in a CDU may need to be every twelve, or even eight hours, as the optimal duration of stay for observation patients is <24 hours. Further study is also needed with respect to the flow of patients through the CDU to identify opportunities for operational improvement.

This study indicates that dedicating case management and physician advisor resources to observation patients may not be an effective or efficient way of decreasing the LOS for observation patients.

## Conclusions

This study suggests that placing patients in a single unit is an effective way to decrease LOS. This study indicates that dedicating case management and physician advisor resources to observation patients may not be an effective or efficient way of decreasing the LOS for observation patients.
